# Trojan horses and tunneling nanotubes enable α-synuclein pathology to spread in Parkinson disease

**DOI:** 10.1371/journal.pbio.3001331

**Published:** 2021-07-20

**Authors:** Santhanasabapathy Rajasekaran, Stephan N. Witt

**Affiliations:** Department of Biochemistry & Molecular Biology, Louisiana State University Health Sciences Center, United States of America

## Abstract

In Parkinson’s disease, Lewy bodies form in the gut or nose and spread into the midbrain. This Primer explores the implications of a study indicating that this spread is due to lysosomes “infected” with prion-like α-synuclein transmitting from cell-to-cell via tunneling nanotubes.

## Teaser

In Parkinson disease (PD), Lewy bodies (LBs) form in the gut or nose and spread into the midbrain. A study in this issue indicates that the spread is due to lysosomes “infected” with prion-like alpha-synuclein (α-syn) transmitting from cell to cell via tunneling nanotubes (TNTs).

Parkinson disease (PD) is due to the progressive degeneration of dopaminergic neurons in the midbrain, and the affected neurons contain inclusions called Lewy bodies (LBs) and Lewy neurites (LNs) that are filled with aggregates and amyloid fibers of the presynaptic protein alpha-synuclein (α-syn) [1]. In a classic study, Braak showed that LB/LN pathology develops in the enteric nervous system of PD patients, and he hypothesized “that a putative environmental pathogen capable of passing the gastric epithelial lining might induce α-syn misfolding and aggregation in specific cell types of the submucosal plexus and reach the brain via a consecutive series of projection neurons [2,3].” Li later found that neurons grafted into 2 PD patients were found 11 to 16 years later, when the patients died, to contain LBs, suggesting that the LBs had spread from host to graft [4]. These 3 studies and others [5,6] have led the field to the current paradigm, i.e., a gut-related insult triggers α-syn to form cytotoxic, prion-like conformers that then spread along the gut–brain axis. How α-syn spreads is a mystery begging to be unraveled. In this issue, Dilsizoglu Senol and colleagues [7] focus on the role of lysosomes and tunneling nanotubes (TNTs) in spreading pathogenic conformations of α-syn.

**Fig 1:**
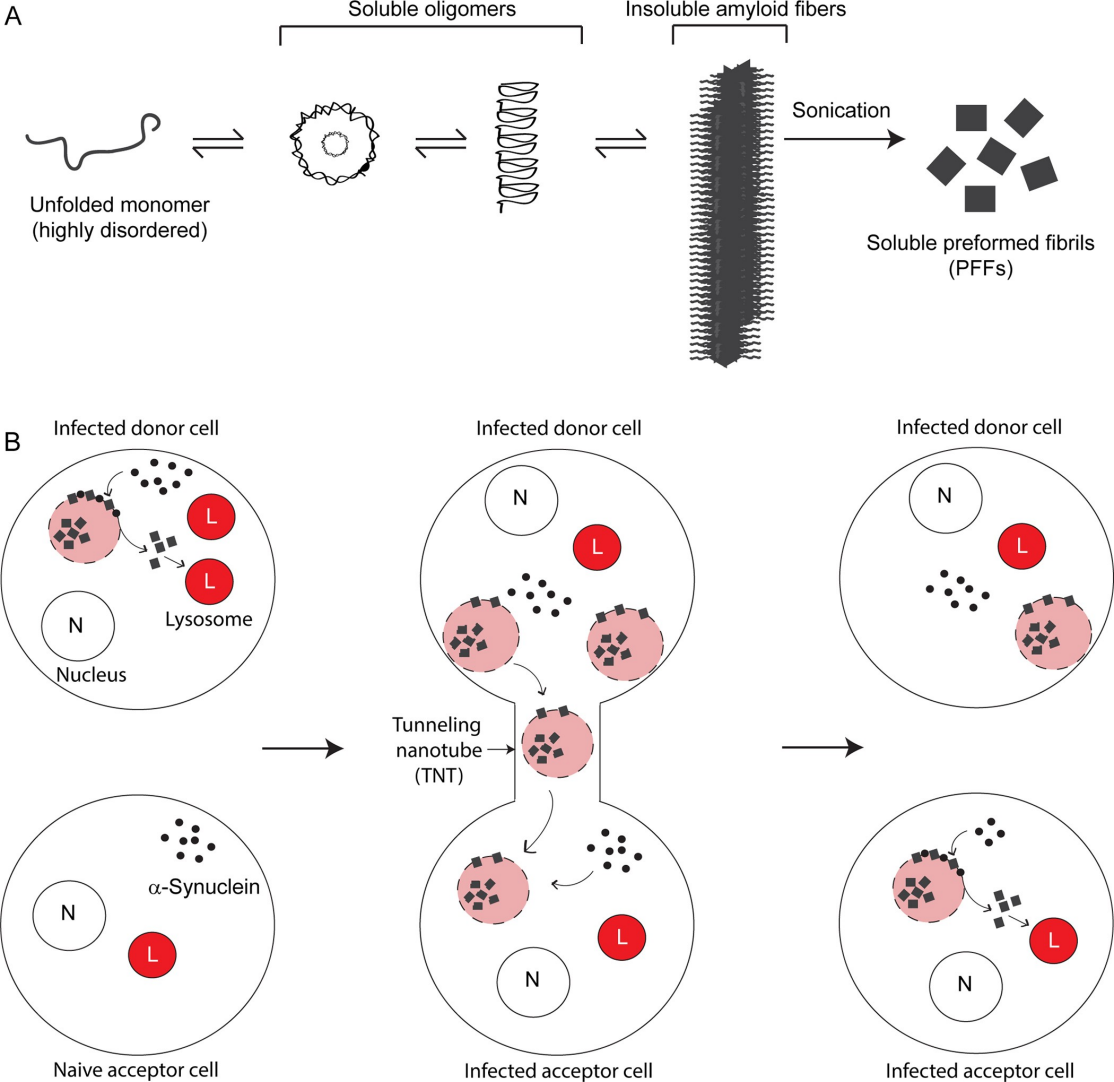
Cell-to-cell transmission via TNTs of dysfunctional lysosomes infected with pathogenic α-syn. **(A)** The myriad states of α-syn. Monomeric α-syn, depending on the milieu, self-associates into soluble oligomers, and, ultimately, insoluble amyloid fibers. PFFs are made by first generating amyloid fibers (shaking for 7 days at 37°C), washing, resuspending, and sonicating. PFFs are characterized for size and concentrations and are typically 50 nm in length. Adding a cysteine residue to the carboxyl terminus of α-syn facilitates labeling PFFs with a fluorophore. PFFs are toxic to cells. **(B)** Dysfunctional lysosomes transmit from cell to cell. The donor cells were undifferentiated mouse catecholaminergic neuronal (CAD) cells. CAD cells take up fluorescently tagged PFFs from the cell culture medium, and these particles enter the lysosomes. PFFs damage the lysosomes: Infected lysosomes double in size, have higher pH, have decreased activity of lysosomal proteases, and are leaky relative to uninfected lysosomes (left images). The infected lysosomes redistribute to the cell periphery, increasing the probability that they tunnel out of the cells through nanotubes. Once in a naive acceptor cell, a unique surface reaction takes place: PFFs embedded in the membrane of the infected lysosome catalyze the conversion of endogenous α-syn monomers into prion-like particles. α-syn, alpha-synuclein; CAD, Cath.a–differentiated; L, lysosome; N, nucleus; PFF, preformed fibril; TNT, tunneling nanotube; λ, α-syn monomer; ■, PFF.

The α-syn protein is composed of 3 domains: an amphipathic domain (residues 1 to 60); the hydrophobic NAC domain (non-Aβ component of Alzheimer disease plaques, residues 61 to 95); and an acidic tail (residues 96 to 140). The monomer, which is intrinsically unfolded, tends to aggregate and fibrillize (Fig 1A). In vitro, amyloid fibers of α-syn are prepared by incubating α-syn monomers with shaking for several days. Sonication breaks the long fibers into short soluble amyloid seeds, which are referred to as preformed fibrils (PFFs) (Fig 1A). PFFs can be injected into mice or added to cells in culture.

In previous work, the Abounit group showed that lysosomes filled with α-syn PFFs spread intercellularly by TNTs [8]. TNTs are made of F-actin, have diameters of 50 to 800 nm and lengths of up to several cell diameters, and allow for the selective transfer of organelles [9,10]. In their study in this issue [7], Dilsizoglu Senol and colleagues analyzed the effects of fluorescently tagged PFFs on the recipient cell’s lysosome morphology, function, leakiness, and spatial distribution using super-resolution microscopy, transmission electron microscopy, and several other techniques. Imaging revealed that PFFs interact with lysosomes in different ways: Some are luminal, whereas others are surface bound. For cells incubated with PFFs, there was a significant increase in both the size and leakiness of lysosomes “infected” with PFFs, but no change in lysosome number compared to untreated controls cell. A curious change also occurred in the distribution of lysosomes: Cells preferentially shifted their leaky, dysfunctional α-syn–laden lysosomes from the perinuclear region to the periphery compared to untreated control cells (Fig 1B), and this was not accompanied by an increase in lysosomal exocytosis. Small interfering RNA (siRNA)-mediated knockdown of *TFEB*, which is the transcription factor that controls lysosomal biogenesis, abolished this shift. Shifting infected lysosomes to the cell periphery increases the probability that one will stumble (or perhaps be escorted) into a nanotube tunnel and tunnel out of the donor into the naive acceptor cell, and this is exactly what was captured by various techniques.

A key finding is that leaky infected lysosomes (aptly referred to as “Trojan horses”) are a platform for prion proliferation because their membranes are embedded with PFFs. Thus, whether the infected lysosomes are in donor or acceptor cells, α-syn monomers collide with and stick to the surface of the infected lysosomes; consequently, the surface-embedded PFFs induce the conversion of surface-bound α-syn molecules into toxic, prion-like molecules (Fig 1B). These newly formed prion-like molecules then enter infected or uninfected lysosomes, which ultimately transfer through TNTs to seed pathology in healthy neighboring cells.

In addition to nanotubes, release of α-syn from cells can occur by secretion or shedding of exosomes and uptake via pinocytosis or receptor-mediated endocytosis, respectively. All of these mechanisms are being explored, and a cell surface receptor that binds to α-syn fibrils has even been identified [11]. A challenge is to show that nanotubes form in neurons along the gut–brain axis, but it is hard to imagine that neurons will lack such connections. If the mechanistic details of how lysosomes are transferred through TNTs can be deciphered, then one strategy will be to search for molecules that inhibit such transfer. Inhibiting the formation of TNTs is another possibility, although this could produce unintended consequences. The pathological spread could also be thwarted by identifying drugs that tightly bind to α-syn prions. Such drugs would abolish the proliferation of amyloid in cells. Ultimately, reliable biomarkers of the disease must be found so that the disease can be treated long before pathology spreads to the brain. Drugs that inhibit cell-to-cell transfer or that block prion proliferation would be a brilliant way to halt the progression of the disease.

## Abbreviations

α-syn: alpha-synuclein
LB: Lewy body
LN: Lewy neurite
NAC: non-Aβ component of Alzheimer disease plaques
PD: Parkinson disease
PFF: preformed fibril
siRNA: small interfering RNA
TNT: tunneling nanotube

## References

[1] Spillantini MG, Schmidt ML, Lee VM, Trojanowski JQ, Jakes R, Goedert M, et al. Alpha-synuclein in Lewy bodies. Nature. 1997;388(6645):839–40. doi: 10.1038/42166 . 9278044

[2] Braak H, Del Tredici K, Rub U, de Vos RAI, Steur E, Braak E. Staging of brain pathology related to sporadic Parkinson’s disease. Neurobiol Aging. 2003;24(2):197–211. doi: 10.1016/s0197-4580(02)00065-9 . 12498954

[3] Braak H, Vos RAI, Bohl J, Tredici K. Gastric alpha-synuclein immunoreactive inclusions in Meissner’s and Auerbach’s plexuses in cases staged for Parkinson’s disease-related brain pathology. Neurosci Lett. 2006;396(1):67–72. doi: 10.1016/j.neulet.2005.11.012 . 16330147

[4] Li J-Y, Englund E, Holton JL, Soulet D, Hagell P, Lees AJ, et al. Lewy bodies in grafted neurons in subjects with Parkinson’s disease suggest host-to-graft disease propagation. Nat Med. 2008;14(5):501–3. doi: 10.1038/nm1746 . 18391963

[5] Luk KC, Kehm V, Carroll J, Zhang B, O’Brien P, Trojanowski JQ, et al. Pathological alpha-synuclein transmission initiates Parkinson-like neurodegeneration in nontransgenic mice. Science. 2012;338(6109):949–53. doi: 10.1126/science.1227157 . 23161999 PMC3552321

[6] Kim S, Kwon SH, Kam TI, Panicker N, Karuppagounder SS, Lee S, et al. Transneuronal Propagation of pathologic alpha-synuclein from the gut to the brain models Parkinson’s disease. Neuron. 2019;103(4):627–41. doi: 10.1016/j.neuron.2019.05.035 . 31255487 PMC6706297

[7] Dilsizoglu Senol A, Samarani M, Syan S, Guardia CM, Nonaka T, Liv N, et al. α-Synuclein fibrils subvert lysosome structure and function for the propagation of protein misfolding between cells through tunneling nanotubes. PLoS Biol. 2021;19(7):e3001287. doi: 10.1371/journal.pbio.3001287 PMC8291706 34283825

[8] Abounit S, Bousset L, Loria F, Zhu S, de Chaumont F, Pieri L, et al. Tunneling nanotubes spread fibrillar alpha-synuclein by intercellular trafficking of lysosomes. EMBO J. 2016;35(19):2120–38. doi: 10.15252/embj.201593411 . 27550960 PMC5048354

[9] Rustom A, Saffrich R, Markovic I, Walther P, Gerdes HH. Nanotubular highways for intercellular organelle transport. Science. 2004;303(5660):1007–10. doi: 10.1126/science.1093133 . 14963329

[10] Sartori-Rupp A, Cervantes DC, Pepe A, Gousset K, Delage E, Corroyer-Dulmont S, et al. Correlative cryo-electron microscopy reveals the structure of TNTs in neuronal cells. Nat Commun. 2019;10(1):342. doi: 10.1038/s41467-018-08178-7 . 30664666 PMC6341166

[11] Mao XB, Ou MT, Karuppagounder SS, Kam TI, Yin XL, Xiong YL, et al. Pathological alpha-synuclein transmission initiated by binding lymphocyte-activation gene 3. Science. 2016;353(6307). doi: 10.1126/science.aah3374 . 27708076 PMC5510615

